# Cost-Effectiveness of Extending the National Influenza Vaccination Program in South Korea: Does Vaccination of Older Adults Provide Health Benefits to the Entire Population?

**DOI:** 10.3390/vaccines10060932

**Published:** 2022-06-10

**Authors:** Gyeongseon Shin, Daewon Kang, Hee Jin Cheong, Sang-Eun Choi

**Affiliations:** 1 College of Pharmacy, Korea University, Sejong City 30019, Korea; gyeongseon4265@gmail.com (G.S.); dwkang85@gmail.com (D.K.); 2 Division of Infectious Diseases, Department of Internal Medicine, Korea University College of Medicine, Gurodong-ro 148, Seoul 08308, Korea; heejinmd@korea.ac.kr

**Keywords:** influenza, vaccination, cost-effectiveness, economic evaluation, adults aged 50–64

## Abstract

The South Korean government has successfully improved influenza vaccination coverage for individuals aged 65 years or older as part of its National Immunization Program (NIP). Those aged 50–64 years without funded vaccination care have significantly lower vaccination rates and face a substantial risk of influenza-related complications. We use a dynamic epidemiological and economic model to investigate the cost-effectiveness of expanding the universal vaccine fund to include those aged 50–64. The epidemiological model is estimated using the susceptibility-infection-recovery model and influenza and influenza-like illness incidence rates, which were calculated by the National Health Insurance Service–National Sample Cohort from the 2008/09 to 2012/13 influenza seasons but excluding the 2009/10 season for pandemic influenza A (H1N1). The decision tree economic model is assessed from societal and healthcare sector perspectives. The proposed policy would eliminate 340,000 annual influenza cases and prevent 119 unnecessary deaths. From a societal perspective, the proposed policy would reduce costs by USD 68 million. From a healthcare perspective, the cost is USD 4318 per quality-adjusted life years. Within the study range, sensitivity analyses found consistent cost-effectiveness results. The influenza vaccine for adults aged 50–64 appears to be cost-saving or cost-effective and, thus, should be considered for the NIP.

## 1. Introduction

According to the World Health Organization (WHO), seasonal influenza epidemics cause between 3 and 5 million severe cases and 290,000–650,000 fatalities per year worldwide [1]. In South Korea, influenza results in 2900excess deaths [2] and a socioeconomic burden of USD 43 million annually [3]. Vaccination is the most effective way to prevent influenza, particularly in high-risk groups such as the elderly, children, and those with chronic diseases [4]. Getting vaccinated is important for both efficacy, effectiveness, and cost-effectiveness [5,6,7].

In many countries, routine influenza vaccinations are recommended and supported for people older than 60 or 65. Furthermore, several countries have recently lowered the recommended vaccination age because of the increasing prevalence of chronic diseases [8,9]. For example, the Advisory Committee on Immunization Practices in the United States dropped the recommended age for universal immunization from 65 to 50 [10]. In the United Kingdom, all persons older than age 50 are included in the National Immunization Program (NIP) [11,12]. The Korean government also recommends vaccination for those older than age 50 [13], with free vaccination for those 65 years or older. However, recommendations alone are insufficient to improve vaccination rates without direct funding, such as free vaccinations or reimbursements [14]—knowledge reflected in the coverage rate. Currently, the vaccination rate is 83% for those 65 years or older, but only 35% for those ages 50–64 [15]. Adults older than 65 have mostly agreed that vaccination is a preventative strategy [16], but people ages 50–64 need special attention because they tend to have lower vaccine acceptance [17].

Several studies have demonstrated that vaccination in healthy adults is increasingly efficacious and cost-saving [18,19,20]. Furthermore, a growing number of studies have demonstrated that a universal vaccination program for those aged 50–64 can be cost-saving [21,22] or cost-effective [8,23,24,25].

In Korea, the effect of the policy to extend vaccination to people over 50 years of age has been studied using a static model [26]. However, a static model cannot sufficiently explain the indirect effect [27]. The indirect effect is known to as herd immunity, which provides indirect protection to an individual who has not been vaccinated [28]. The proposed policy must be accurately evaluated to ensure the appropriate use of limited health service budgets. Adequate immunization can indirectly protect unvaccinated individuals in the community and significantly affect the strategy’s estimated cost-effectiveness [29]. A dynamic model would be superior for assessing herd immunity.

This paper uses a dynamic influenza transmission model to evaluate the cost-effectiveness of expanding a vaccine policy implemented in Korea to include the population 50 years and older to account for both indirect protection of the unimmunized through herd immunity and direct protection of the immunized.

## 2. Methods

This cost-effectiveness study used a dynamic transmission model to predict the effects of expanding universal vaccination for individuals aged 50–64 years on influenza-related outcomes (prevalence, hospitalizations, and deaths). Korea’s demographic distribution shows that the 2020 population is represented by seven distinct age groups: 1–6, 7–12, 13–18, 19–49, 50–59, 60–64, and ≥65 years. The following framework was adopted to compare the cost-effectiveness of the current and expanded two vaccination strategies.

(1) Target population of current NIP
  · Children aged 6 months to 12 years
  · Pregnant women
  · Adults aged 65 years or more
(2) Target population of proposed policy
  · Current target population
  · Individuals aged 50–64 years

The assumption is that those not covered by the above strategies will be self-paid vaccinated. The current vaccination strategy reflects the 2020/21 season in Korea. Vaccination is based on age and supported for pregnant women, who are assumed to be included in the vaccination rate by age. Exceptions were made for groups that received temporary support based on concerns over a possible COVID-19 twindemic.

### 2.1. Model Framework

The model is divided into two parts: epidemiologic and economic. The former was analyzed using a dynamic model to reflect the characteristics of infectious diseases. The epidemiologic effect of the expansion of national vaccination was simulated using the susceptibility–infection–recovery (SIR) model [30,31,32], following the population as age-structured SIR. The dynamics of influenza infection at the population level were simulated using a deterministic transmission model. Although this study focused on the impact of increased vaccination on individuals aged 50–64 years, the study subjects were represented in the aforementioned seven groups for the population aged more than one year to understand the impact on society, including social transmission through contact and indirect herd protection/immunity effects. Each time, the transmission coefficient was revised to obtain the target basic reproduction number (R_0_) for the specified length of infectiousness.

The epidemic model was calibrated to adjust for age group susceptibility and infection rates. Before starting the immunization program, the model was calibrated using illness and demography data. The force of infection was determined using least squares techniques to fit the model to the data on age-specific prevalence [33].

**Figure 1:**
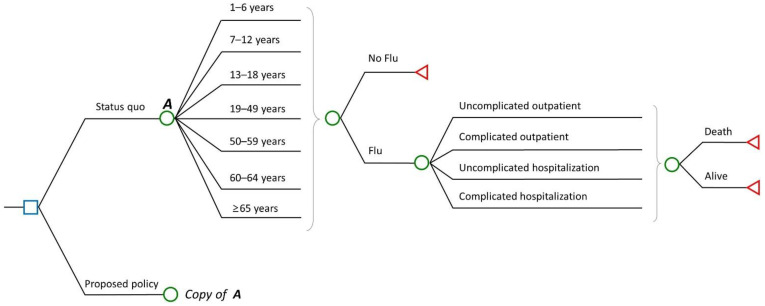
Economic model structure.

The economic model estimates the probabilistic cost and utility for various situations that may occur through the decision tree model strategy (Figure 1). This model estimates the probabilistic cost and utility of several situations to examine the public health effects and cost-effectiveness of influenza vaccinations with greater coverage among those in Korea aged 50–64 years relative to those without NIP. All subjects are assigned to the decision tree, and the derived cost and utility are estimated individually. In the epidemiological simulation, the number of people infected with influenza by age was estimated using the National Health Insurance Service–National Sample Cohort (NHIS–NSC) [34]. The NHIS–NSC is a group that could represent South Korea in consideration of social & economic variables (location of residence, age, income rank, etc.), and 2% of the population was selected. The incidence of complications and hospitalization rates obtained from the NHIS-NSC were included in the model. In the economic model—unlike in the epidemiologic model—the probability values were assumed to not change within the study period and were applied the same. Each probability branch was assumed to be mutually exclusive.

### 2.2. Parameters and Data

#### 2.2.1. Vaccine Parameters

Every individual in the simulation is assumed to be vaccinated with a quadrivalent inactivated vaccine (QIV) because the trivalent inactivated vaccine (TIV) used for NIP was replaced with QIV beginning with the 2020/21 season. The first QIV was approved in Korea in 2014; however, the sample cohort data used for the study are from 2008 to 2013 (the 2009/10 influenza season is excluded as a pandemic). Therefore, the incidence data obtained from the NHIS–NSC result from TIV inoculation. An adjustment was made to the epidemiological model, to assume that all vaccinations were QIV.

The effect of the QIV was estimated by including the impact of the vaccine added by matching B to the effect of the TIV [35]. In the comparative strategy, the vaccination coverage of individuals aged 50–64 years was assumed to be 80%, and the Korea National Health & Nutrition Examination Survey (KNHANES) 2018–2019 [15] was used for other age groups (Table 1).

#### 2.2.2. Disease Burden and Health Outcomes

Prevalence can be determined from influenza cases in the NHIS–NSC and influenza laboratory surveillance data. Both influenza and influenza-like illness (ILI) cases were considered. Ideally, laboratory confirmation of influenza should have been performed on all ILI or respiratory infections but is frequently impracticable because of resource restrictions, and individuals with influenza-associated illnesses may not be tested [44]; therefore, using only the influenza ICD-10 codes (J09, J10, J11) can cause underestimation [45]. However, because ILI cases may include other respiratory viral infections, actual influenza cases are estimated by multiplying the weekly incidence rate of laboratory-confirmed influenza from WHO’s FluNet [37] and the Korea Centers for Disease Control and Prevention (KCDC) [46]. This method was used in WHO’s manual for estimating the influenza disease burden [44] and previously published studies [26,45]. The number of influenza cases for each strategy was calculated by reflecting the 2020 population structure in the ILI rate.

Various health outcomes related to influenza—outpatient visits, complications, hospitalizations, and deaths—were modeled (ICD-10 codes for complications are in Supplementary Table S1) [26,47]. These probabilities were obtained from the NHIS–NSC, and influenza outbreaks and results were determined using an epidemiological model for each strategy (Table 2). Influenza-related complications and deaths are limited to those occurring within four weeks of diagnosis. Cases that used medical facilities for acute influenza complications (in Supplementary Table S1) one month prior to the announcement of the influenza epidemic by the Korea Disease Control and Prevention Agency (KDCA) were excluded from influenza-related complications.

#### 2.2.3. Utilities

Table 2 shows a summary of utility data. Because an influenza-related utility study has not yet been conducted in South Korea, we have referred to previous studies. The results were derived from a two-round modified Delphi survey that included seven infectious disease specialists [18]. Disutilities of 3.8 days [41,42] and five days were applied for outpatients without complications and outpatients with complications, respectively. For hospitalizations, a loss of utility equal to the number of days of hospitalization was applied regardless of complications. The baseline utility of adults measured using EQ-5D from KNHANES [15] was applied, and the remainders were estimated to have a utility weight of 1.

### 2.3. Costs

The model considered both direct and indirect costs from societal and healthcare sector perspectives (Table 3). Direct costs were primarily medical expenses per person for ILI obtained from the NHIS–NSC and immunization data. Medication costs were excluded from NHIS–NSC claim data and anti-influenza agents (Oseltamivir), and rapid antigen test costs were added to prevent overestimation. However, because antibiotics can be prescribed together when complications occur, they are included in the total amount of claims. In addition, transportation and nursing expenses were calculated using KNHANES data.

Indirect costs were based on the cost of absenteeism and premature death from illness. The median wage and labor participation rate by age were considered. The absence period was applied differently depending on the condition of the disease. The KDCA recommends that people diagnosed with influenza do not go to work and return to work when their body temperature is maintained without a fever reducer. Therefore, outpatients without complications were assumed to lose 3.8 workdays [41,42]—the infection period applied to the epidemiological model, and outpatients with complications lost 5 workdays. For hospitalization, absenteeism was applied for the length of hospitalization. Additionally, a human capital approach was used to measure the costs of premature death [50]. The expected production of those who died prematurely equaled the loss of society and was affected by age-specific wages and economic participation [26,45]. All costs in the model were converted to 2020 costs using the consumer price index [51]. Furthermore, we discounted future costs at the 4.5 percent rate recommended by the South Korean Ministry of Economy and Finance as a social discount rate [52].

### 2.4. Sensitivity and Scenario Analyses

Various one-way sensitivity analyses were performed to examine the uncertainty of the critical input parameters and scenario analyses were performed to determine the differences according to this study’s hypothesis and analytical strategy. The base case values of vaccine parameters, cost, quality-of-life weights (utility), and discount rate were varied in a one-way deterministic sensitivity analysis (DSA) and a probabilistic sensitivity analysis (PSA). In addition, the scenario included an analytical perspective, indirect effect, vaccination strategy, and coverage.

## 3. Results

### 3.1. Base Case

According to the epidemiological model, approximately 3.59 million cases of influenza are reported each year, with 277,063, 313,803, and 2094 cases of complications, hospitalizations, and deaths, respectively (Table 4). Under the proposed policy, nearly 5.7 million more people aged 50–64 years would be vaccinated. Increasing the number of people vaccinated could reduce influenza infections by approximately 35% for those 50–64 years of age and 9.5% for all age groups. In addition, this proposed policy reduces influenza complication cases by 6.6%, hospitalizations by 6.8%, and deaths by 5.7% at the population level.

Incremental cost-effectiveness ratios (ICERs) are calculated for the base case and various scenarios (Table 4 and Table 5). The base case proposed policy, which targets individuals 50–64 years of age, dominates the status quo and saves USD 68 million.

### 3.2. Scenario Analyses

All scenarios are cost-saving strategies from a societal perspective (Table 5). In contrast, it was just a cost-saving approach for the age group scenario in the healthcare sector. A scenario that targeted only individuals aged 60–64 years, with the possibility of a partial policy, was explored because a vaccination policy requires a significant budget. This strategy has become dominant because the additional vaccination cost is relatively modest for individuals aged 60–64 years, given a greater vaccination rate than for individuals aged 50–59 years from both perspectives. The indirect effect had the most significant impact on ICER from a societal perspective; however, the proposed policy was dominant and showed high cost-effectiveness from a healthcare sector perspective (6465 USD/ quality-adjusted life year (QALY).

### 3.3. Sensitivity Analyses

**Figure 2:**
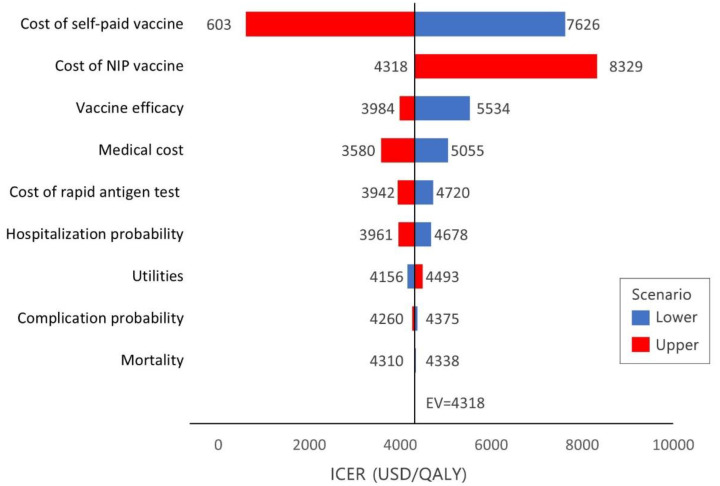
Tornado diagram of deterministic sensitivity analysis (healthcare sector perspective). Tornado diagram showing an increase (red) or decrease (blue) of other parameters for the lower and upper limits of the healthcare perspectives; EV, expected value; ICER, incremental cost-effectiveness ratio; QALY, quality-adjusted life years.

A tornado diagram depicts the results of the DSAs (Figures S1–S4). In the base case, from the societal perspective, cost-saving results were obtained in all DSAs. Although cost-effectiveness indicated consistent results, vaccine prices were the most sensitive to the ICER of the healthcare sector (Figure 2). Other variables had a negligible impact on the ICER. The supplementary figure provides more information on the effects of parameter modifications on the various scenario results.

**Figure 3:**
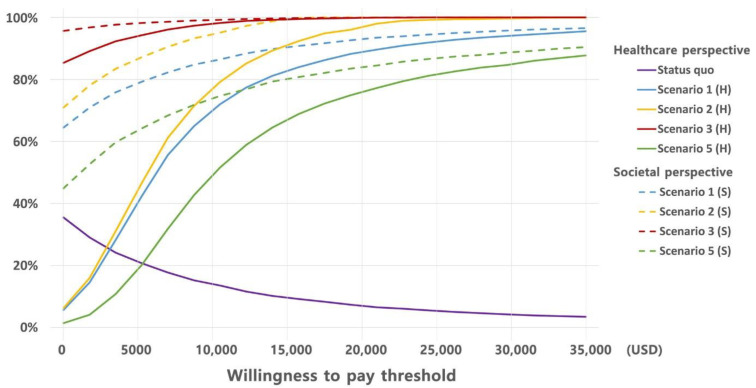
Cost-effectiveness acceptability curve by scenario: Scenario 1 (H) represents the base case from a healthcare sector perspective, Scenario 2 (H) represents 60% coverage for individuals aged 50–64 years from a healthcare sector perspective, Scenario 3 (H) includes only individuals aged 60–64 years in the policy from a healthcare sector perspective, Scenario 5 (H) has no indirect effects from a healthcare sector perspective, Scenario 1 (S) represents the base case from a societal perspective, Scenario 2 (S) represents 60% coverage for individuals aged 50–64 years from a societal perspective, Scenario 3 (S) includes only individuals aged 60–64 years in the policy from a societal perspective, Scenario 5 (S) has no indirect effects from a societal perspective. The threshold of willingness-to-pay is USD 30,681 per quality-adjusted life years.

A cost-effectiveness acceptability curve and plane are shown as PSA results (Figure 3). The PSA results are robustly distributed across the cost-effectiveness plane. At a willingness to pay of USD 30,681 per QALY, which is approximately the average per capita gross domestic product in Korea, the acceptability curve shows that the proposed policy has an 85.7% chance of being cost-effective from a healthcare sector perspective.

## 4. Discussion

Several countries have recently considered lowering the recommended age for influenza vaccination from 64 years to 50 [8,9,10,11,12]. Given this trend, this study considered expanding the NIP in Korea, which successfully increases immunization rates beyond reducing the cost burden associated with self-paid vaccines. This study found that the cost for adults aged 50–64 years was USD 136 million for self-paid vaccinations and an additional cost is expected USD 99 million for increasing coverage. Still, the approach provides a USD 167 million benefit from a societal perspective and costs approximately USD 4318 per QALY gained from a healthcare sector perspective.

Vaccinating children, the elderly, and pregnant women is generally cost-saving or cost-effective. However, the evidence for immunizing adults has been inconsistent and highly dependent on factors such as geographic location, vaccination effectiveness, and the value of lost output [53]. Therefore, the comparison between countries may be restricted, and the inclusion of NIP for individuals aged 50–64 years should be examined when considering national and social conditions. In Korea, the free vaccination policy is highly effective. Those aged 65 years and above who were eligible for free vaccination from NIP had an 83% vaccination rate, compared with 35% for those aged 50–64 who were not. In addition, compared with other countries that provide free vaccines for ages 65 and above, such as the United Kingdom (72%), New Zealand (62%), and Australia (56.2%), Korea has a high immunization rate [54]. As a result, vaccine policies in Korea may be higher than in other countries.

Several studies have analyzed the expansion of NIP in Korea [45,55,56] but were based on static models and failed to account for the effect of indirect protection (herd immunity), which could consequently underestimate the impact of vaccinations. Only Choi et al. [26] included Koreans aged 50–64 years. Although that study found a trend similar to ours, the benefit obtained in our study was significant. Choi et al. found that vaccinating adults aged 50–64 years with TIV was cost-saving, and the QIV was cost-effective (3661 USD/QALY) from a societal perspective. In contrast, from a healthcare sector perspective, both TIV and QIV were not cost-effective (ICER = 37,352 USD/QALY, 86,463 USD/QALY, respectively). However, in our study, both TIV and QIV were cost-saving from a societal perspective and even cost-effective (4318 USD/QALY) from a healthcare sector perspective. These differences can be found in the indirect effects. Because the previous study disregarded the benefit of indirect effects, they are likely to be underestimated.

Our fundamental assumption is that by providing free vaccines from the NIP, vaccination rates will reach 80%. In recent years, South Korea has successfully extended NIP over the past few years, so it is reasonable to assume that the proposed policy will also be successful. For example, vaccination for children under 12 years old was gradually extended from 2016 to 2018, and the coverage rate increased dramatically from 61.2% in 2014 to 83.1% in 2019. In addition, when free vaccination was made available to pregnant women in 2019, the vaccination rate increased from 36.5% in 2014 to 68.5% in 2019 [57]. The effect of the free vaccination policy is not limited to South Korea. A Cochrane review compared free immunization vouchers to paid invitations in two randomized controlled trials. Free vaccination vouchers increased vaccination rates by 2.36 (95% CI 1.98–2.82; p 0.001) [58]. However, it is not clear how successful the NIP extending will increase vaccination rate. Assuming a vaccination rate of 80% may be overly optimistic, so we performed a sensitivity analysis to determine whether it is cost-effective even at lower immunization rates (Scenario 2 in Table 5 and Figure 3). As a result, we confirmed that the proposed policy is still cost-saving or cost-effective even when the inoculation rate is 60%. This result is even more cost-effective than 80% coverage, as vaccination expenditures are decreased. This study, therefore, provides insight into the possible success of the extending of vaccination policy.

This study confirmed that extending NIP may provide individual and social benefits. This is consistent with cost-effectiveness studies in other countries [8,21,22,23,24,25]. Furthermore, regardless of the perspective, this study indicated that vaccination in the group aged 60–64 years will offer benefits in both cost and utility. It can be proposed that a phased introduction of the group aged 60–64 years first in the policy extension will be effective.

This study is the first to use a dynamic epidemiologic model to analyze influenza vaccination cost-effectiveness in Korea. The dynamic epidemiologic model can reflect the infectious disease characteristics of seasonal influenza. Moreover, considering social contact patterns showed that the spread of disease from contact between age groups and the indirect effect of vaccinating some age groups reflected the impact on the entire population. The indirect effect shown in this study increased the cost per QALY by 1.5 times, and the actual effect of vaccinations can be much more significant than expected, which is observed through the vaccination coverage and efficacy resulting from the indirect effect [59].

This study has several limitations. First, limitations exist from the limitations of the data. The influenza data were only used for four seasons: 2008/09 through 2012/13 (with 2009/10 excluded as a pandemic). The analysis should consider year-to-year variations in the influenza disease burden; ideally, more than five years of data are required [60]. However, because of data limitations, instead of including only four seasons in the study, a sensitivity analysis of influenza variation and vaccine effects was performed using previous studies and recent monitoring data. Another limitation of the data is the lack of studies on social contact patterns in Korea. Because the contact pattern determines the size of the epidemic, understanding the contact pattern among individuals to estimate the spread of infectious respiratory diseases, such as measles and influenza, is critical [61]. Ibuka et al. [43] investigated social contact patterns in Japan, and Japan was also used in domestic measles research because the country has a similar sociodemographic structure to Korea [61]. The accuracy of future analyses can be improved by using domestic social contact pattern research.

The second limitation is the uncertainty in estimating the prevalence of ILI and influenza-related diseases. Analysis using claim data requires some assumptions and operational definitions because identifying all clinical symptoms, such as a high temperature, is challenging. For this reason, the incidence of influenza is calculated using ILI, which carries the risk of overestimation. This study addressed that issue by estimating the actual influenza epidemic levels by applying the detection rate of influenza viruses among respiratory viruses from WHO’s FluNet and KCDC to the estimated ILI epidemic size. Determining the exact incidence rate of influenza-related complications is also challenging [62,63]. In addition to respiratory diseases—the most common complications of influenza—only acute complications related to encephalitis, myositis, myocarditis, pericarditis, stroke, and myocardial infarction were included. Therefore, complications may have been underestimated in the elderly.

Finally, the impact of COVID-19 on influenza is unclear. It is now well accepted that wearing a mask and avoiding personal contact, for example, social distance, is essential for infection prevention. It causes post-pandemic behavioral changes, and these impacts may persist in the future. More research is needed to determine the social contact as well as disease and vaccination characteristics.

## 5. Conclusions

Our study model is one of the first to analyze the health and economic impacts of seasonal influenza vaccinations of people aged 50–64 years. The findings show that relative to the present policy, the NIP for adults aged 50–64 years will be cost-saving from a societal perspective and cost-effective (ICER = 4318 USD/QALY) from a healthcare sector perspective. The scenario that excluded the indirect effect had the most significant influence on ICER, and—relative to previous studies—indirect effects had significant impacts. Therefore, analysis using dynamic transmission models could be helpful; however, additional research is needed to obtain an accurate picture of indirect effects.

## Figures and Tables

**Table 1:** Population and vaccination parameters.

Variables	Value	Source/Comment
Population by age group		[36]
1–6	2,297,013	
7–12	2,799,426	
13–18	2,843,464	
19–49	22,532,473	
50–59	8,645,014	
60–64	3,950,469	
≥65	8,496,077	
Immunization rate of influenza vaccine by age group		
*Current*		[15]
1–6	0.862	
7–12	0.702	
13–18	0.310	
19–49	0.267	
50–59	0.284	
60–64	0.498	
≥65	0.826	
*Proposed policy*		Assumption
50–59	0.800	
60–64	0.800	
Effectiveness of vaccine by age group		Calculated with reference to Capri et al. [35] using WHO data [37] and Cochrane review [38,39,40]
1–6	0.694 (0.640–0.950)	
7–12	0.711 (0.640–0.923)	
13–18	0.711 (0.640–0.924)	
19–49	0.655 (0.590–0.901)	
50–59	0.655 (0.590–0.901)	
60–64	0.655 (0.590–0.901)	
≥65	0.634 (0.580–0.900)	
Transmission coefficient by age group	0.450	Fitting
Recovery rate	0.263	Inverse of the infectious period [41,42]
Population mixing matrix between age group	See Ref [43].	Japanese contact matrix was used

**Table 2:** Probabilities and utilities.

	Age Group							Range of DSA	Source ^a^
	1–6	7–12	13–18	19–49	50–59	60–64	≥65		
Probability									
Influenza case	0.370	0.106	0.051	0.041	0.057	0.069	0.085	–	N,I
Complication/influenza case	0.130	0.071	0.043	0.024	0.041	0.064	0.122	80–120%	N
Hospitalization/influenza case	0.176	0.076	0.062	0.020	0.050	0.080	0.109	–	N
Death ^b^/influenza case	0.564	0.000	0.767	1.958	5.731	28.425	389.981	–	N
Utility weight (decrement)									
Baseline utility	1 ^c^	1 ^c^	1 ^c^	0.973	0.957	0.943	0.887	80–120%	[15]
Uncomplicated outpatient	−0.35	−0.35	−0.35	−0.35	−0.35	−0.35	−0.35	80–120%	[45]
Complicated outpatient	−0.4	−0.4	−0.4	−0.4	−0.4	−0.4	−0.4	80–120%	[45]
Uncomplicated hospitalization	−0.4	−0.4	−0.4	−0.4	−0.4	−0.4	−0.4	80–120%	[45]
Complicated hospitalization	−0.5	−0.5	−0.5	−0.5	−0.5	−0.5	−0.5	80–120%	[45]

^a^ The data calculated using National Health Insurance Service–National Sample Cohort are labeled “N”, and the data calculated using influenza laboratory surveillance are labeled “I”,. ^b^ Deaths per million. ^c^ Assumption; DSA, deterministic sensitivity analysis.

**Table 3:** Cost inputs by age group.

	Age Group	Base Case	SE	Range of DSA	Source ^a^
Cost (USD)					
*Vaccination*					
NIP vaccine dose cost ^b^	1–12	25.69	–	25.69–38.56	[48]
	50–64	23.45	–	23.45–35.18	Assumption
	≥65	23.45	–	23.45–35.18	[48]
Self-paid vaccine ^c^	All	31.01	0.10	22.72–39.51	[49]
*Direct medical cost*					
Uncomplicated outpatient ^c^	1–6	73.71	0.29	80%–120%	N
	7–12	69.36	0.27	80%–120%	N
	13–18	66.44	0.23	80%–120%	N
	19–49	58.28	0.08	80%–120%	N
	50–59	59.03	0.16	80%–120%	N
	60–64	60.09	0.28	80%–120%	N
	≥65	60.24	0.18	80%–120%	N
Complicated outpatient ^c^	1–6	128.44	0.51	80%–120%	N
	7–12	119.06	0.46	80%–120%	N
	13–18	115.95	0.40	80%–120%	N
	19–49	118.32	0.17	80%–120%	N
	50–59	119.38	0.32	80%–120%	N
	60–64	113.63	0.53	80%–120%	N
	≥65	108.11	0.32	80%–120%	N
Uncomplicated hospitalization ^c^	1–6	525.19	2.07	80%–120%	N
	7–12	563.54	2.17	80%–120%	N
	13–18	590.60	2.02	80%–120%	N
	19–49	669.92	0.96	80%–120%	N
	50–59	883.46	2.34	80%–120%	N
	60–64	1027.54	4.81	80%–120%	N
	≥65	1018.42	3.01	80%–120%	N
Complicated hospitalization c	1–6	830.34	3.27	80%–120%	N
	7–12	773.68	2.98	80%–120%	N
	13–18	663.93	2.28	80%–120%	N
	19–49	950.77	1.36	80%–120%	N
	50–59	956.59	2.53	80%–120%	N
	60–64	1648.05	7.71	80%–120%	N
	≥65	1198.25	3.54	80%–120%	N
Anti-influenza agents d	1–6	8.08	–	–	H
	7–12	14.50	–	–	H
	≥13	14.65	–	–	H
Rapid antigen test^c^	All	24.69	0.15	15.31–31	[49]
*Direct nonmedical cost* (USD)					
Nursing^c^	All	36.41	1.15	80%–120%	[15]
Transportation for outpatient ^c^	All	3.11	0.03	3.04–3.18	[15]
Transportation for hospitalization ^c^	All	14.65	0.58	13.30–15.99	[15]
*Indirect cost*					
Cost per working day lost (USD) ^e^	19–49	74.87	–	80%–120%	S
	50–59	92.41	–	80%–120%	S
	60–64	51.00	–	80%–120%	S
Duration of treatment (days)					
Uncomplicated outpatient ^d^	All	3.8	–	–	[41,42]
Complicated outpatient ^d^	All	5	–	–	Assumption
Hospitalization without complication ^c^	1–6	5.9	0.015	–	N
	7–12	5.3	0.034	–	N
	13–18	5.5	0.033	–	N
	19–49	7.3	0.019	–	N
	50–59	9.4	0.020	–	N
	60–64	9.6	0.056	–	N
	≥65	9.2	0.019	–	N
Hospitalization with complication ^c^	1–6	6.8	0.064	–	N
	7–12	5.9	0.325	–	N
	13–18	5.4	0.120	–	N
	19–49	8.1	0.127	–	N
	50–59	8.5	0.140	–	N
	60–64	12.3	0.151	–	N
	≥65	8.8	0.140	–	N

^a^ The data calculated using National Health Insurance Service–National Sample Cohort are labeled “N”, the data calculated using Health Insurance Review & Assessment Service 2020 annual weighted average price information are labeled "H", and the data calculated using Statistics Korea’s employment and labor statistics data are labeled “S”. ^b^ Triangular distribution (base-150%). ^c^ Gamma distribution. ^d^ Fixed variable. ^e^ Triangular distribution (±20%); SE, Standard error; DSA, deterministic sensitivity analysis; NIP, National Immunization Program.

**Table 4:** Base case results of status quo and adding vaccination of those aged 50–64 years in Korea.

	Status quo	Proposed Policy	Difference
Coverage			
Number of people vaccinated	22,281,539	27,940,972	5,659,433
Influenza outcomes			
Total influenza cases	3,587,330	3,245,781	−341,549
Total complications	277,063	258,735	−18,328
Total hospitalizations	313,803	292,613	−21,190
Total influenza-associated deaths	2094	1975	−119
Costs (USD)			
Vaccinations	616,940,439	716,297,174	99,356,735
Outpatient visits	435,557,031	402,499,966	−33,057,065
Hospitalizations	262,794,280	242,647,477	−20,146,803
Direct non-healthcare costs	247,350,909	231,521,717	−15,829,192
Productivity costs	687,038,979	589,043,080	−97,995,899
Health effects			
Total quality-adjusted life years	50,017,614	50,028,303	10,689

**Table 5:** Cost and incremental cost-effectiveness ratio for base case and various scenarios.

Strategy	Age Group	Vaccination Coverage (%)	Indirect Effects	Costs (Million USD)		QALYs	ICER (USD/QALY)	
				Societal	Healthcare		Societal	Healthcare
Status quo	–	–	Yes	2250	1315	50,017,614	–	–
Scenario 1	50–64	80	Yes	2182	1361	50,028,303	dominant	4318
Scenario 2	50–64	60	Yes	2207	1328	50,023,090	dominant	2295
Scenario 3	60–64	80	Yes	2199	1302	50,019,438	dominant	dominant
Scenario 4	–	–	No	2250	1315	50,017,614	–	–
Scenario 5	50–64	80	No	2211	1375	50,026,855	dominant	6465

Notes: The threshold of willingness-to-pay is USD 30,681 per quality-adjusted life years and scenario 1 is the base case; ICER, incremental cost-effectiveness ratio; QALYs, quality-adjusted life years.

## Data Availability

The data presented in this study are available within the article or in the Supplementary Materials.

## Supplementary Materials

The following supporting information can be downloaded at: www.mdpi.com/article/10.3390/vaccines10060932/s1. Figure S1: Tornado diagram of deterministic sensitivity analysis (societal perspective): 50 to 64 years old with vaccine coverage 80%, including indirect effects; Figure S2: Tornado diagram of deterministic sensitivity analysis (without indirect effects): healthcare sector perspective, 50 to 64 years old with vaccine coverage 80%, not including indirect effects; Figure S3: Tornado diagram of deterministic sensitivity analysis (60to 64 years old): healthcare sector perspective, 60 to 64 years old with vaccine coverage 80%, including indirect effects; Figure S4: Tornado diagram of deterministic sensitivity analysis (vaccine coverage 60%): healthcare sector perspective, 50 to 64 years old with vaccine coverage 60%, including indirect effects; Table S1: List of ICD-10 codes used in the definition.
