# Supplementary material for: Cost-Effectiveness of Extending the National Influenza Vaccination Program in South Korea: Does Vaccination of Older Adults Provide Health Benefits to the Entire Population?
Source: Vaccines (Basel). 2022 Jun 10;10(6):932. doi: 10.3390/vaccines10060932 (PMC9228362; doi:10.3390/vaccines10060932)
Supplement: Supplementary file 1 [file vaccines-10-00932-s001.zip › vaccines-1736175-supplementary.pdf]

Supplementary Table S1. List of ICD-10 codes used in the definition.

Definition		ICD-10 codes	Source
Influenza and influenza-like illness (ILI)	Influenza	J09, J10, J11	[1]
	Acute upper respiratory infection	J06	[2]
	Acute lower respiratory infection	J22	
	Pneumonia, organism unspecified	J18	
	Viral infection, unspecified site	B34	
Acute complications related to influenza	Pneumonia	J12, J13, J14, J15, J16, J17, J18	[1]
	Encephalitis	A85.8, A86, A87.8, A87.9, A89, B94.1, G038, G039, G04.0, G04.8, G04.9, G05.1, G05.8, G36	
	Myositis	M60.0, M60.1, M60.8, M60.9	
	Myocarditis, pericarditis	I41.1, I51.4, I30, I31.9, I40, I51.8, B33.2	
	Acute myocardial infarction	I21, I23, I24	
	Stroke	I63	
	Rhabdomyolysis	M62.8	
	Transverse myelitis	G37.3, G37.8, G37.9	

1. Choi, M.J.; Shin, G.; Kang, D.; Lim, J.-O.; Kim, Y.-K.; Choi, W.S.; Yun, J.-W.; Noh, J.Y.; Song, J.Y.; Kim, W.J. Cost-Effectiveness of Influenza Vaccination Strategies in Adults: Older Adults Aged≥ 65 Years, Adults Aged 50–64 Years, and At-Risk Adults Aged 19–64 Years. *Vaccines* **2022**, *10*, 445.
2. Moore, K.; Black, J.; Rowe, S.; Franklin, L. Syndromic surveillance for influenza in two hospital emergency departments. Relationships between ICD-10 codes and notified cases, before and during a pandemic. *BMC Public Health* **2011**, *11*, 1-7.

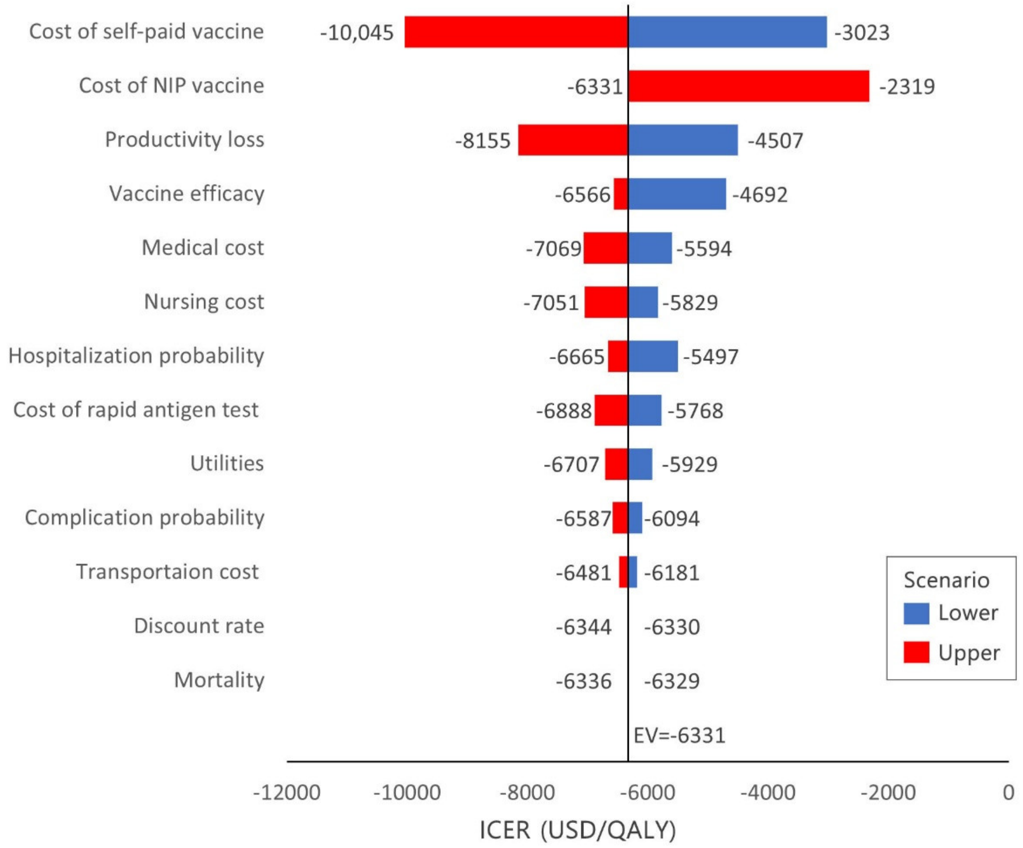

Supplementary Figure S1. Tornado diagram of deterministic sensitivity analysis (societal perspective): 50 to 64 years old with vaccine coverage 80%, including indirect effects.

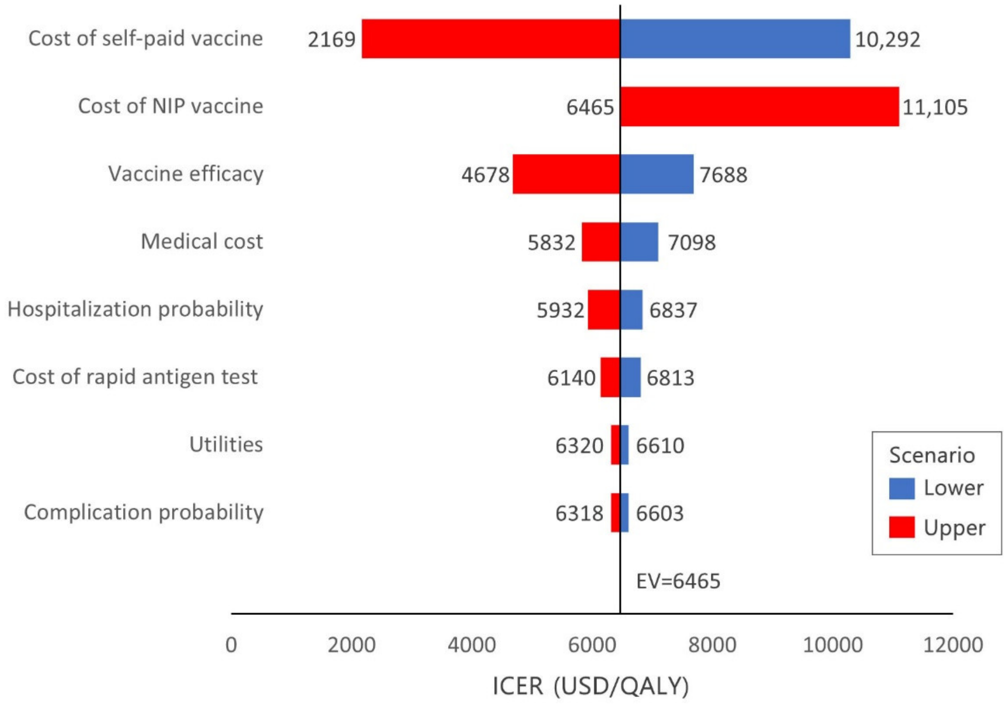

Supplementary Figure S2. Tornado diagram of deterministic sensitivity analysis (without indirect effects): healthcare sector perspective, 50 to 64 years old with vaccine coverage 80%, not including

indirect effects.

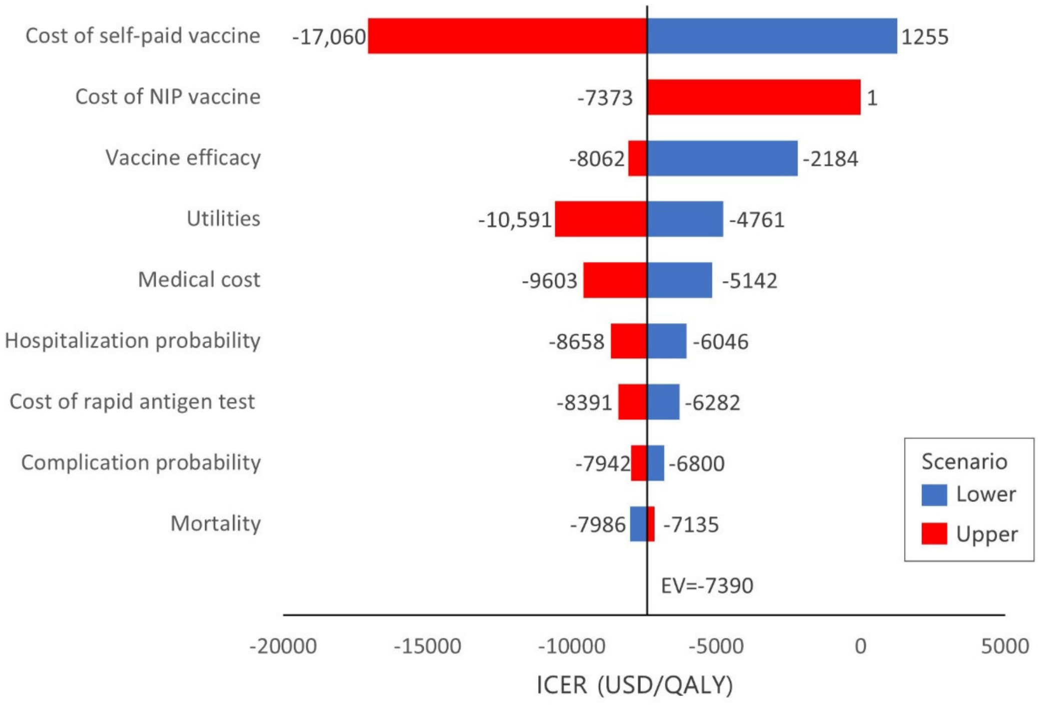

Supplementary Figure S3. Tornado diagram of deterministic sensitivity analysis (60 to 64 years old): healthcare sector perspective, 60 to 64 years old with vaccine coverage 80%, including indirect effects.

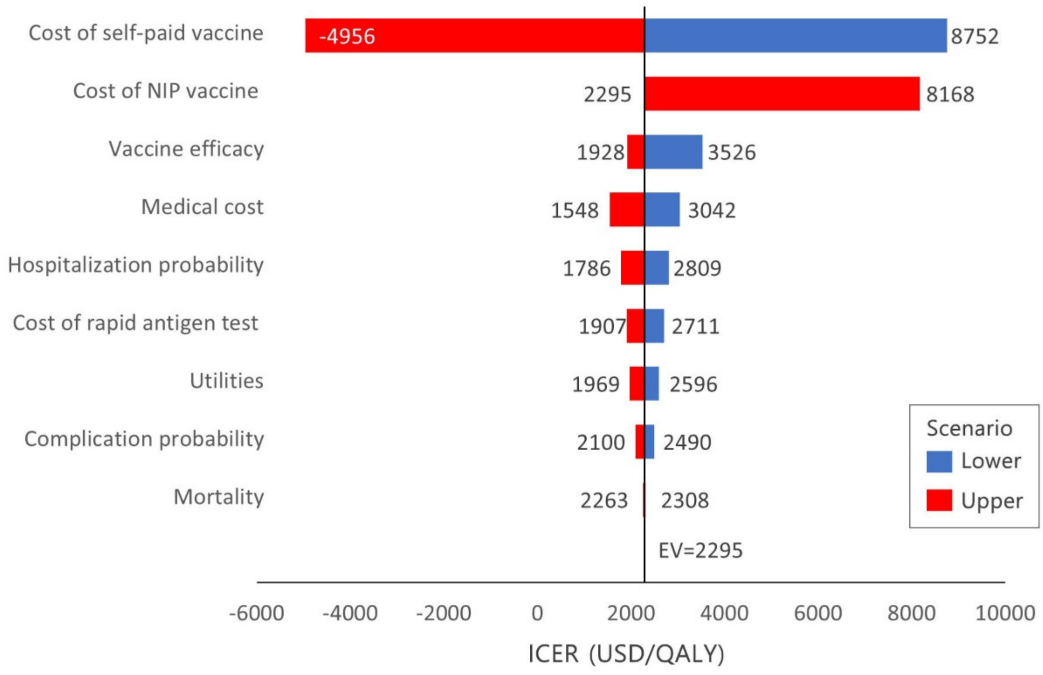

Supplementary Figure S4. Tornado diagram of deterministic sensitivity analysis (vaccine coverage 60%): healthcare sector perspective, 50 to 64 years old with vaccine coverage 60%, including indirect effects.
